# Comparison of florfenicol depletion in dairy goat milk using ultra-performance liquid chromatography with tandem mass spectrometry and a commercial on-farm test

**DOI:** 10.3389/fvets.2022.991772

**Published:** 2022-08-29

**Authors:** Emily D. Richards, Richard V. Pereira, Jennifer L. Davis, Joan D. Rowe, Maaike O. Clapham, Scott E. Wetzlich, Benjamin A. Rupchis, Lisa A. Tell

**Affiliations:** ^1^Food Animal Residue Avoidance and Depletion Program, Davis, CA, United States; ^2^Department of Medicine and Epidemiology, School of Veterinary Medicine, University of California-Davis, Davis, CA, United States; ^3^Department of Population Health and Reproduction, School of Veterinary Medicine, University of California-Davis, Davis, CA, United States; ^4^Department of Biomedical Sciences and Pathobiology, Virginia-Maryland College of Veterinary Medicine, Blacksburg, VA, United States; ^5^Department of Animal Science, University of California-Davis, Davis, CA, United States

**Keywords:** florfenicol, goat, extra-label drug use, drug residue, milk residue

## Abstract

Florfenicol is a broad-spectrum antibiotic commonly prescribed in an extra-label manner for treating meat and dairy goats. Scientific data in support of a milk withdrawal interval recommendation is limited to plasma pharmacokinetic data and minimal milk residue data that is limited to cattle. Therefore, a rapid residue detection test (RRDT) could be a useful resource to determine if milk samples are free of drug residues and acceptable for sale. This study compared a commercially available RRDT (Charm^®^ FLT strips) to detect florfenicol residues in fresh milk samples from healthy adult dairy breed goats treated with florfenicol (40 mg/kg subcutaneously twice 4 days apart) with quantitative analysis of florfenicol concentrations using ultra-performance liquid chromatography with tandem mass spectrometry (UPLC-MS/MS). In addition, storage claims for testing bovine milk using the RRDT were assessed using stored goat milk samples. Milk samples were collected every 12 h for a minimum of 26 days. Commercial RRDT strips remained positive in individual goats ranging from 528 to 792 h (22–33 days) after the second dose, whereas, UPLC-MS/MS indicated the last detectable florfenicol concentration in milk samples ranged from 504 to 720 h (21–30 days) after the second dose. Results from stored milk samples from treated goats indicate that samples can be stored for up to 5 days in the refrigerator and 60 days in the freezer after milking prior to being tested with a low risk of false-negative test results due to drug degradation. Elevated somatic cell counts and bacterial colony were noted in some of the milk samples in this study, but further study is required to understand the impact of these quality factors on RRDT results.

## Introduction

Florfenicol is a broad-spectrum antibiotic approved by the Food and Drug Administration (FDA) for use in cattle, swine, and fish, and by the European Medicines Agency (EMEA) for use in cattle, sheep, swine, and fish. The EMEA has extrapolated maximum residue limits (MRLs) to all food-producing species, including goats, due to the limited number of medicinal products approved for use in minor animal species (1). Despite the florfenicol FDA- and EMEA-approvals for use in ruminants, neither agency has approved florfenicol for use in lactating animals, which results in extra-label use of florfenicol in lactating cattle and small ruminants, even though there is no tolerance (TOL) or MRL established for milk and pharmacokinetic data in milk is limited to a few small studies in cattle (2–4).

In the United States, the Animal Medicinal Drug Use Clarification Act (AMDUCA) permits veterinarians with a valid veterinarian-client-patient-relationship to prescribe FDA-approved medications in an extra-label manner (5). One condition of AMDUCA requires the veterinarian to determine a ‘substantially extended’ withdrawal interval (WDI) based on scientific evidence for extra-label drug use (ELDU) in food-producing species to ensure food products are free of drug residues. The Food Animal Residue Avoidance Databank (FARAD) is a federally funded program that serves to help veterinarians by recommending scientifically-based WDIs following extra-label drug use. According to FARAD internal WDI request data, florfenicol was the most-requested antimicrobial drug for goat meat and milk WDIs between 2015 and 2020. The majority of requests were for WDIs following subcutaneous administration with approximately half of the total submissions requesting milk WDIs. Determining a substantially extended milk WDI is challenging because there is only one published study in lactating dairy cattle following the subcutaneous administration of florfenicol, which reported a 60 h milk half-life and concentrations above the limit of detection up to 588 h after a single 40 mg/kg dose (2).

Given the difficulty of determining a withdrawal interval due to the paucity of florfenicol residue milk data and the consequence of lost product & revenue in the event of antibiotic detection in the bulk tank, rapid residue detection tests provide a useful resource for producers to quickly determine if milk samples are free of drug residues and acceptable for sale. Rapid residue detection tests (RRDT) for detecting florfenicol in raw commingled cow milk are available. The RRDT that detects florfenicol and thiamphenicol is a rapid one step immunoreceptor assay that utilizes lateral flow technology where florfenicol or thiamphenicol interact with colored beads in the lateral flow test strip, leading to presence of colored lines in the test and control zones if residue is not detected, as well as color intensity changes as the florfenicol or thiamphenicol concentrations approach the sensitivity (6). According to the RRDT manufacturer's instructions, the test detects florfenicol or thiamphenicol down to 1 ppb in cow milk stored at 0–7°C and has a specificity of 95%. However, it has been reported that these rapid residue detection tests may be cross-reactive both to similar medications or components of the milk (i.e. somatic cells, bacteria, fat-content, etc.) (7–9) resulting in false positives. The RRDT manufacturer's instructions indicate that there are no interferences in detection from somatic cells at ≤10^6^ SCC/ml or bacteria at ≤ 3 × 10^5^ CFU/ml, but high fat samples (>6.5%) may cause invalid results. Additionally, other amphenicols are the only known medication interferences that are cross-reactive at 100 ppb.

Previous studies evaluating rapid residue detection tests for goat milk have reported that milk secretory mechanisms and milk composition vary between cows and goats, which may affect RRDT results when used with goat milk (8–10). The primary objective of this study was to compare a commercially available RRDT for florfenicol residues in fresh goat milk samples (dosing regimen of 40 mg/kg subcutaneously twice 4 days apart) with quantification of drug residues using ultra-performance liquid chromatography with tandem mass spectrometry. Secondary objectives were to assess the impacts of sample storage prior to testing and potential factors that could result in false positives for the RRDT.

## Materials and methods

### Animal enrollment

The University of California Institutional Animal Care and Use Committee (IACUC) approved all experimental procedures conducted with animals for this study (IACUC Protocol Number 21671). The study was conducted at the University of California Davis goat facility, which utilizes farm practice managements common to those observed at other dairy goat farms in California. Animals enrolled in the study were selected by convenience, based on their lactation and kidding dates. Throughout the sampling period, study does were housed at the University of California, Davis Goat Teaching & Research Facility in penned areas with other does. Goats were fed alfalfa hay twice a day, 3–3.5 lbs 14% dairy ration, and provided water *ad libitum*.

Five lactating does of various breeds (Saanen, *n* = 2, Alpine, *n* = 1, LaMancha, *n* = 1, Alpine-LaMancha cross, *n* = 1), age (range 2–5 years) and weights (range 77.5–113 kg, mean 94.4 kg) were enrolled following a physical examination that included assessment of temperature, pulse, respiration rate, rumen contractions, body condition score, Faffa Malan Chart (FAMACHA) test, and udder palpation, conducted by a single veterinarian (JDR); animals had to demonstrate no apparent clinical disease to be enrolled in the study. All does were administered two subcutaneous 40 mg/kg doses of florfenicol (Nuflor^®^ 300 mg/ml, Merck Animal Health, Madison, NJ, USA) 4 days apart. Does were weighed prior to initial florfenicol administration and the total dose was administered at two injections sites to limit no more than 10 ml being administered at each site, as recommended by the label; injections were administered subcutaneously using an 18 g × 1 inch needle on opposite sides of the body in the region of the abdomen.

### Milk collection

Goats were milked by barn staff twice daily, at ~12 h intervals. A 0.5% iodine teat-dipping solution was used for pre- and post-dipping of teats; after application, teats were dried with paper towels after at least a 40 s contact time. Prior to milk collection, each teat was stripped twice and fore-milk was examined for abnormal milk. Milk was collected in a clean glass jar and transferred to a clean metal bucket until each milking was complete. Milk was weighed using a Dairy Herd Information Association (DHIA)-certified hanging scale and mixed at least 3 times by pouring milk between two buckets. Samples were immediately transferred to 3 or 5 ml cryovials, which were kept at ambient temperature until RRDT was completed (maximum 30 min). At specified time points, additional 15–30 ml aliquots of milk were collected in 15 or 30 ml Eppendorf tubes for additional sampling or tests. Further details are described in the ‘Milk Quality and Component Sampling’ and ‘Antibiotic Residue Screening of Stored Milk Samples’ sections.

### Milk quality and component sampling

**Figure 1:**
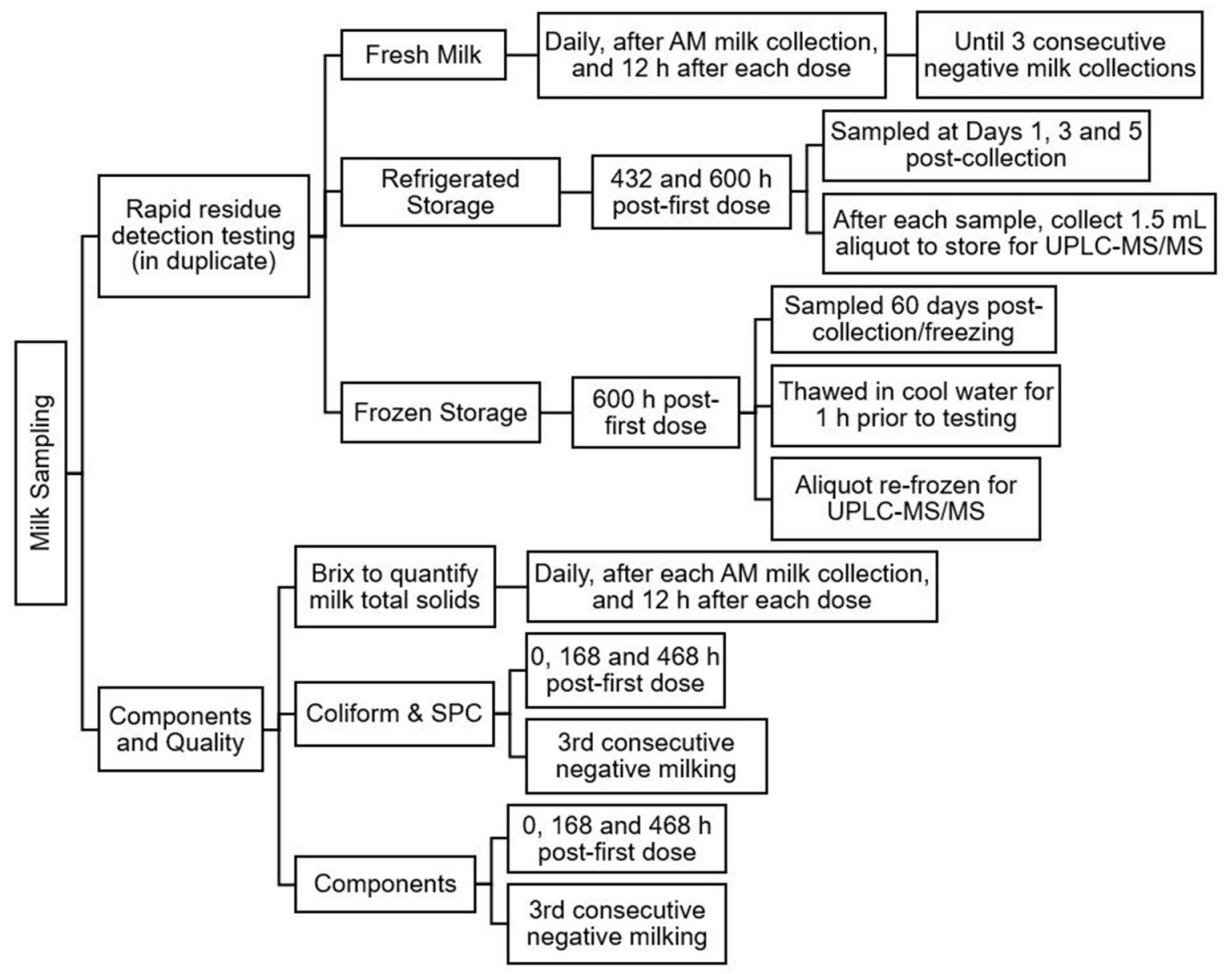
Overview of the milk sampling protocol following treatment of lactating does with florfenicol 40 mg/kg subcutaneously twice, 4 days apart.

Figure 1 provides a brief overview of the milk sampling protocol.

For rapid quantification of milk total solids in the individual goat milk samples, a Brix test was completed on each morning milk sample and 12 h after each florfenicol dose. This was done by inverting the Eppendorf tube multiple times, and adding one to three drops of milk to a digital refractometer (Palm Abbe™ model PA202X; MISCO; Solon, OH) using a 1 ml plastic pipet. The refractometer was calibrated daily using standard protocols.

At four time points (0, 168, 468 h and at the final milking) milk was shipped overnight to two accredited milk testing laboratories and tested for components and quality including fat, protein, lactose, solids non-fat (SNF) percent, somatic cell count (SCC, cells/ml), milk urea nitrogen (MUN, mg/dl; Central Counties DHIA, Atwater, CA) and coliform count (CFU/ml), standard plate count (SPC, CFU/ml; Sierra Dairy Labs, Tulare, CA). Milk was transferred to 30 ml tubes and shipped overnight to each laboratory with ice packs. For coliform count testing, milk was placed in tubes without preservative, while the samples tested for components and quality were placed in tubes with bronopol 18% preservative (Bronolab W-II Liquid, Advanced Instruments, Norwood, MA). Some of the samples were collected and stored in the refrigerator for up to 72 h prior to shipping due to pre-designated collection times.

### Antibiotic residue screening of fresh milk samples

Fresh milk samples were tested for residues using a commercial RRDT (Charm^®^ FLT; Charm Sciences Inc., Lawrence, MA). Strips were stored, handled and utilized according to the manufacturer's instructions and individual samples were tested in duplicate. Once all samples were placed on the incubator, the lid was closed and the timer started. If the test strip results were ambiguous, images of the strip were sent to an additional sample collector for independent evaluation. If a testing strip indicated an invalid result, the milk sample was tested a second time. Milk samples were aliquoted and stored at −20°C until they could be transferred to a −70°C freezer (within 48 h), where they were maintained prior to ultra-performance liquid chromatography with tandem mass spectrometry (UPLC-MS/MS) analysis. This procedure was completed on morning milk samples starting on day 0, as well as the first evening milk sample after each dose, then continued daily on the morning milk samples until the strips, run in duplicate for a single milk sample, were interpreted as negative. If the milk sample run in duplicate was negative, then the stored milk sample from the prior evening milking (which was stored overnight in a refrigerator after mixing) was tested. Milk samples were tested in duplicate until samples from three consecutive milking events were negative. Results for RRDT screening of fresh milk samples are reported as hours or days post-second dose (PSD).

### Antibiotic residue screening of stored milk samples

According to the manufacturer of the RRDT, bovine milk samples can be stored prior to testing in the refrigerator or freezer (<-15°C) for 5 days or 2 months, respectively. To evaluate the potential for storing goat milk prior to testing, ~15 ml of milk was collected at two time points (432 and 600 h, 18 and 25 days, respectively, post-first dose). These time points were chosen based on cattle data (4) that indicated florfenicol was detected in milk ~26 days after subcutaneous administration, therefore would likely guarantee positive results at 432 h (18 days) post-first dose and approach the sensitivity of the RRDT strips at the 600 h (25 days) post-first dose collection. Results for RRDT screening of stored milk samples are reported as hours or days post-first dose (PFD) in order to ensure milk samples would not be confused during later UPLC-MS/MS quantification of all samples.

For testing refrigerated samples, the milk was stored in a standard consumer refrigerator (~0–5°C) then tested in duplicate 1, 3 and 5 days post-collection, with approximately 1 ml aliquots removed concurrently and frozen at −70°C for later UPLC-MS/MS analysis. For the samples stored frozen, approximately a 1.5 ml aliquot was collected and stored in a −20°C freezer for 60 days. On day 60, samples were thawed in cool water for 1 h, shaken and tested in duplicate using the RRDT strips, with the remaining milk sample being re-frozen in a −20°C freezer and transferred as soon as possible to a −70°C freezer until UPLC-MS/MS analysis could be completed.

### Sample analysis/quantification of florfenicol concentrations

Florfenicol and florfenicol amine concentrations in goat milk samples were quantified using UPLC-MS/MS. Our study utilized the UPLC-MS-MS method for measuring florfenicol and florfenicol amine concentrations in milk and milk products from multiple species including goat milk developed by Power et al. (11). The present method was modified to simplify the extraction and reduce the sample volume and solvent usage, while maintaining sensitivity. Power et al. (11) showed sample stability for 12 months at −20°C for both florfenicol and florfenicol amine.

Florfenicol and florfenicol amine reference standards were obtained from a commercial chemical supplier (Cayman Chemical, Ann Arbor, MI). Florfenicol-d3 (Fd3) and florfenicol amine-d3 (FNd3) were used as isotopically labeled internal standards and were also obtained from a commercial chemical supplier (Toronto Research Chemicals, New York, ON, Canada). Stock solutions for florfenicol, florfenicol amine, florfenicol-d3, and florfenicol amine-d3 were initially made at a concentration of 1,000 μg/ml by dissolving the dry crystalline solids in 100% methanol. The florfenicol amine standard was supplied as the HCl salt, so the concentrations were corrected in the first step such that all calculated concentrations are shown as the free form of florfenicol amine. The florfenicol and florfenicol amine spiking solutions were combined and further diluted in 100% ACN to form eight spiking standards at concentrations of 0.1, 0.4, 2, 5, 10, 25, 37.5, and 50 μg/ml. The florfenicol-d3 and florfenicol amine-d3 were combined and further diluted in 100% ACN to a concentration of 12.5 μg/ml and added to each standard and sample at a constant volume of 10 μl.

Milk samples were prepared in triplicate by combining 250 μl of milk with 250 μl of 0.1 M phosphate buffer at pH 7, 10 μl of the internal standard addition solution (5 μg/ml of both Fd3 and FNd3 in ACN), and 1.5 ml ethyl acetate (EtOAc) in 2 ml polypropylene (PP) microcentrifuge tubes. The samples were loaded onto a vortex table to extract for 10 min and sample extracts were then centrifuged (Eppendorf Microcentrifuge Model 5415R, Eppendorf North America, Enfield, CT) at 16,100 × g for 5 min. The top organic layer of the resulting supernatant solution was then transferred to clean 4 ml glass vials and dried down under nitrogen at 40°C. Dried residues were then reconstituted by adding 1 ml of 10/90 ACN/H_2_O, capping, and then vortexing again for 10 min. Approximately 500 μl of the reconstituted extracts were transferred and then filtered using syringeless filter vials (Separa^®^, GVS, Bologna, Italy) with a 0.2 μm polyvinylidene fluoride (PVDF) membrane. These were then placed in the refrigerated autosampler (6°C) of the UPLC-MS/MS for analysis.

Sample extracts were subjected to chromatographic separation on a UPLC system with a phenyl column (Waters Acquity UPLC^®^ BEH Phenyl, 100 mm length × 2.1 mm ID × 1.7 μm, Waters Corporation, Milford, MA) and matching guard column (Waters Acquity UPLC^®^ BEH Phenyl VanGuard Pre-Column, 5 mm length × 2.1 mm ID × 1.7 μm, Waters Corporation, Milford, MA) maintained at 40°C. Sample volume was 5 μl. Mobile phase A consisted of 10 mM ammonium acetate (NH_4_Ac) + 0.05% (v/v) acetic acid (HAc) in H_2_O, and mobile phase B consisted of 100% ACN. The mobile phase was delivered to the UPLC column at a flow rate of 0.4 ml per min. The gradient elution program is shown in Supplementary Table 1.

The retention times of florfenicol amine and florfenicol were ~0.88 and 1.89 min, respectively. The UPLC column effluent was pumped directly without any split into a triple-quadrupole mass spectrometer (Waters Xevo TQD, Waters Corporation, Milford, MA) equipped with a Zspray ionization source which was operated in positive-ion electrospray mode (ESI+) for florfenicol amine, and negative-ion electrospray mode (ESI–) for florfenicol with both modes using multiple reaction monitoring (MRM). The parent and product ion transitions for the compounds of interest are shown in Supplementary Table 2. Mass spectrometer parameters used for the detection of florfenicol are shown in Supplementary Table 3.

An eight-point calibration curve made up in blank goat milk was prepared in an identical manner to the samples using a concentration range of 8–4,000 ppb milk for both florfenicol and florfenicol amine. Using these standards, a linear calibration curve was constructed for both analytes to determine the analyte concentration in samples based on the sample:IS ratio. The limit of detection (LOD) and limit of quantitation (LOQ) were established according to the method described by Shah et al. in 1992 (12). The UPLC-MS-MS method was validated according to the FDA Bioanalytical Method Validation Guidance for Industry (13) with the exception of the selection of the highest quality control concentration (the highest quality control was based on the concentration range for milk samples) and a lower limit of quantitation was not established. Validation included spiking control milk at four concentrations (8, 24, 240 and 2,400 ppb). Five replicates of each concentration were analyzed each day for 3 days. The results from these analyses were used to establish precision, accuracy and recovery.

### Statistical analysis

Descriptive analysis was conducted using a commercial spreadsheet program (Microsoft Office Excel, Microsoft Corp., Redmond, WA) and a commercial statistical software (JMP Pro 16.0, SAS Institute Inc., Cary, NC).

Analysis of variance for florfenicol (ppb) and florfenicol amine (ppb) concentrations in frozen samples over time was conducted in the statistical software. Normal distribution was evaluated using Shapiro-Wilk test, and because normality was not met, a non-parametric approach was used. The non-parametric Kruskal-Wallis in the statistical software was used to evaluate a significant difference in the florfenicol (ppb) and florfenicol amine (ppb) distribution by time in days. A *P* value < 0.05 for this analysis indicated that a significant difference in florfenicol concentrations was observed between any day pairwise comparisons.

## Results

### Descriptive data of enrolled animals and milk characteristics

**Figure 2:**
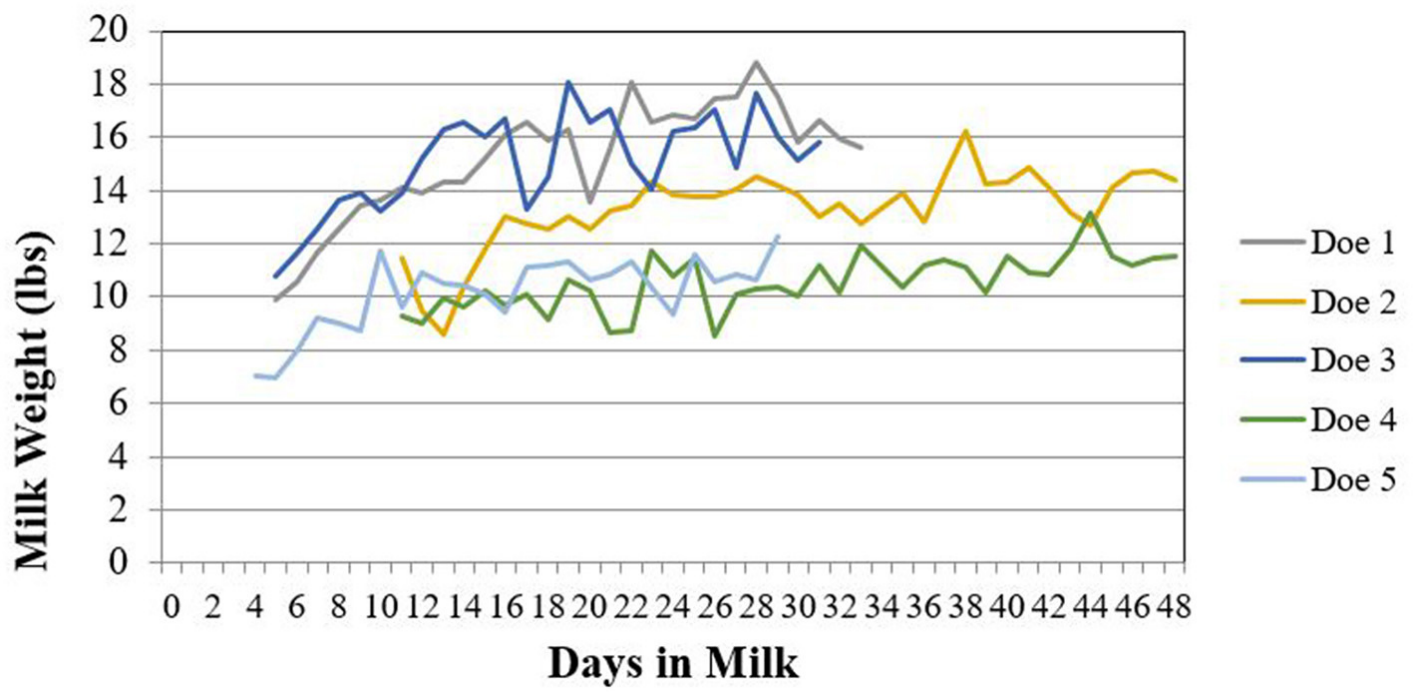
Daily milk production for does (*n* = 5) enrolled in a florfenicol milk residue depletion study. Milk weights were only collected while does were enrolled in the study.

Supplementary Table 4 is a summary of the physical characteristic data for the five enrolled does. This information was recorded during physical examination at enrollment. Does varied from 2 to 5 years of age and body weights ranged from 77.5 to 113 kg. Body condition scores (BCS) ranged from 3 to 3.75 out of 5 and Faffa Malan Chart (FAMACHA^®^) scores ranged from 1 to 2. Milk components and quality results for each time point sampled are summarized in Table 3. The daily milk production for each doe enrolled in the study is shown in Figure 2.

### Method validation

The standard curve for both florfenicol and florfenicol amine was linear with coefficient of determination (*R*^2^) for all curves >0.99. Tables 1, 2 include the florfenicol and florfenicol amine method validation parameters for the UPLC-MS-MS milk method. Analysis of non-spiked control milk showed no interfering peaks at the retention time for both florfenicol and florfenicol amine. The analytical limit of detection (LOD) was ~3 ppb for milk as determined by the signal-to-noise ratio of 3 for both florfenicol and florfenicol amine. The limit of quantification (LOQ) was considered to be the lowest concentration on the linear regression calibration curve at 8 ppb milk for both analytes. Average (±SD) inter assay accuracies were 112.2 ± 2.4, 100.3 ± 2.9, 102.6 ± 1.6 and 108.5 ± 0.7%, respectively for 8, 24, 240 and 2,400 ppb, with an inter assay coefficient of variation of 3.0 ± 1.6 for florfenicol. For florfenicol amine, average (±SD) inter assay accuracies at 8, 24, 240 and 2,400 ppb were 108.8 ± 4.2, 97.2 ± 3.0, 103.8 ± 1.3 and 105.8 ± 1.5%, respectively with an inter assay coefficient of variation of 3.1 ± 1.3%. For florfenicol, intra assay coefficients of variation were 3.6, 4.4, 2.0 and 1.0%, for 8, 24, 240 and 2,400 ppb, respectively and 2.8, 3.1, 1.8 and 1.6%, respectively for florfenicol amine. Average recoveries were 86.0 ± 1.1% for florfenicol and 30.9 ± 0.7% for florfenicol amine.

**Table 1:** Sensitivity, precision and accuracy parameters for the ultra-performance liquid chromatography with tandem mass spectrometry analytical method used to measure florfenicol concentrations in goat milk following florfenicol administration to lactating does.

**Quality control drug**	**Intra-assay variation**	**Inter-assay**	**Accuracy average**	**Recovery**
**concentration (ppb)**	**(RSD) average (range) (%)**	**variation (RSD) (%)**	**(range) (%)**	**(%)**
8	3.6 (2.9–4.2)	3.8	112.2 (109.4–113.6)	84.7
24	4.4 (3.7–4.8)	4.7	100.3 (97.2–102.9)	87.9
240	2.0 (1.8–2.7)	2.3	102.6 (101.4–104.4)	85.4
2,400	1.0 (0.9–1.3)	1.1	108.5 (107.7–109.0)	86.0

**Table 2:** Sensitivity, precision and accuracy parameters for the ultra-performance liquid chromatography with tandem mass spectrometry analytical method used to measure florfenicol amine concentrations in goat milk following florfenicol administration to lactating does.

**Quality control**	**Intra-assay variation**	**Inter-assay**	**Accuracy average**	**Recovery**
**drug concentration (ppb)**	**(RSD) average (range) (%)**	**variation (RSD) (%)**	**(range) (%)**	**(%)**
8	2.8 (2.1–3.5)	4.2	108.8 (106.1–113.6)	31.1
24	3.1 (1.3–5.6)	4.3	97.2 (95.3–100.7)	29.7
240	1.8 (0.7–2.7)	2.1	103.8 (102.5–104.9)	30.1
2,400	1.6 (1.0–2.5)	2.0	105.8 (104.7–107.4)	32.8

**Table 3:** Milk components and quality results for does (*n* = 5) enrolled in a florfenicol milk residue depletion study. Samples were collected at each time point post-first dose^a^.

**Doe**	**Time post-**	**DIM^b^**	**Milk^c^**	**Fat^d^**	**Protein^d^**	**Lactose^d^**	**SNF^e^**	**MUN^f^**	**SCC^g^**	**Coliform^h^**	**SPC^i^**
	**first dose^a^**
1	0	5	5.5	5.25	4.1	4.4	9.1	21.6	152	<10	<1,000
	168	12	7.7	3.9	3.3	4.5	8.5	24.5	35	<10	160,000
	468	31	8.3	3.3	2.6	4.3	7.8	25.4	33	<10	18,000
	696	60	7.6	3.9	2.3	4.3	7.4	25.5	50	<10	27,000
2	0	11	6.6	3.4	3.4	4.4	8.5	22.1	6,379	<10	3,000
	168	18	6.8	3.1	3.1	4.4	8.3	23.8	1,936	<10	7,000
	468	37	6.0	3.4	2.8	4.3	7.9	24.6	4,590	<10	3,000
	888	74	7.8	2.5	2.6	4.4	7.9	28.1	1,365	>1,500	>5,700,000 (est.)
3	0	5	6.0	4.6	4.0	4.4	9.0	23.6	97	>1,500	>5,700,000 (est.)
	168	12	8.6	3.2	3.5	4.5	8.8	29.0	32	<10	<1,000
	468	Sample not
		available
	648	32	6.4	2.2	2.3	4.5	7.7	21.2	50	<10	9,000
4	0	11	5.5	3.6	3.7	4.8	9.3	20.4	721	<10	1,000
	168	Sample not
		available
	468	37	4.8	3.6	2.9	4.6	8.3	25.6	962	<10	<1,000
	888	48	6.3	2.2	2.6	4.7	8.1	26.1	292	<10	58,000
5	0	4	4.4	6.2	5.1	4.5	10.3	9.0	6,312	<10	21,000
	168	11	5.5	5.5	4.1	4.8	9.8	13.1	2,985	<10	11,000
	468	30	5.1	4.4	3.4	4.9	9.1	13.8	2,112	460	11,000
	624	59	6.1	3.3	3.3	5.0	9.2	14.6	872	>1,500	>5,700,000 (est.)

a Time point in hours.

b Days in milk.

c Milk production in lbs. This represents only one milking. The farm milked does twice a day.

d Percent fat, protein and lactose values for milk.

e Percent solids-non-fat value for milk.

f Milk urea nitrogen (mg/dl).

g Somatic cell counts (cell/ml × 1,000).

h Coliform counts in milk (CFU/ml).

i Standard plate counts (CFU/ml).

### Florfenicol and florfenicol amine in milk after treatment

**Figure 3:**
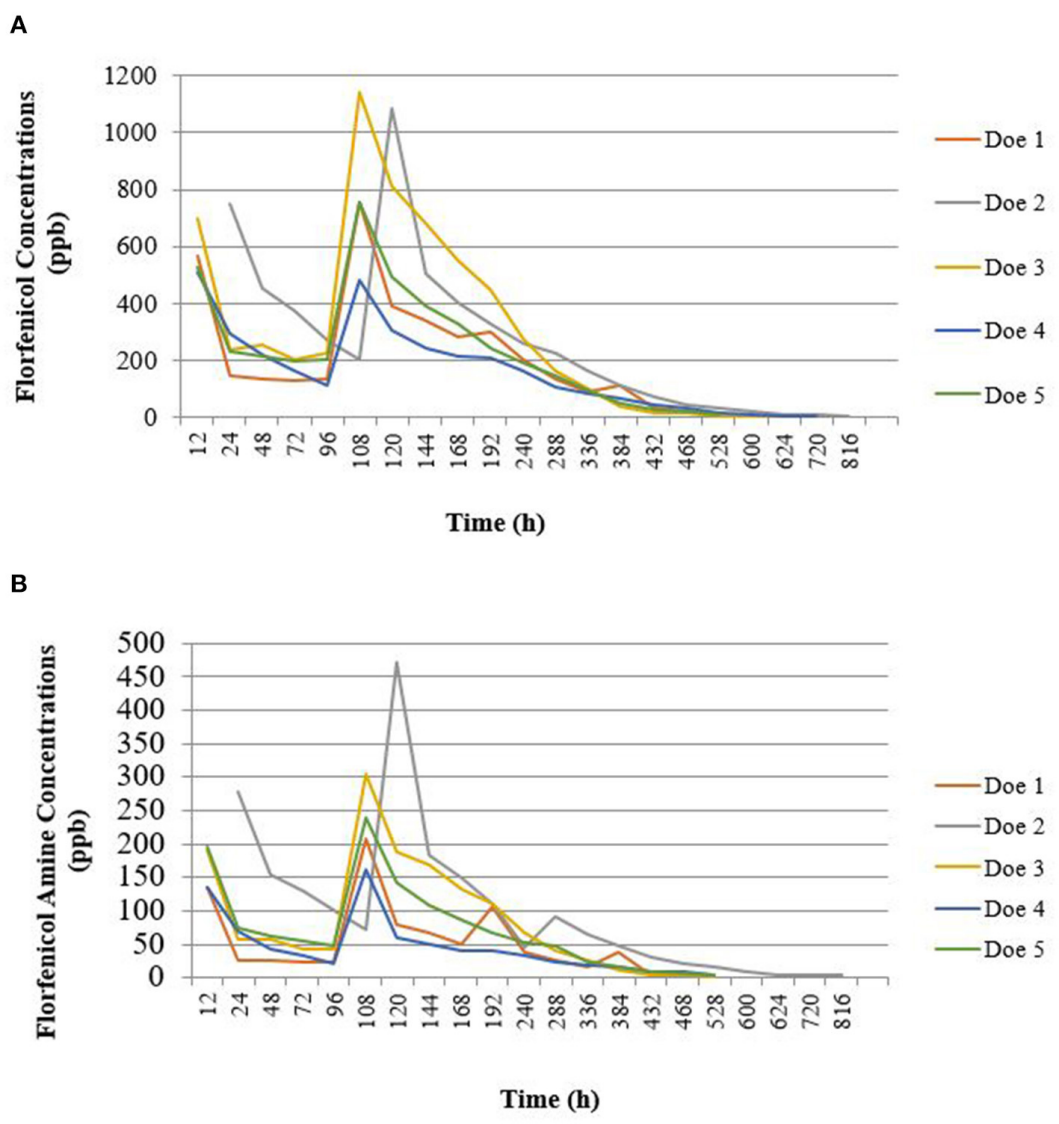
Goat milk sample florfenicol **(A)** and florfenicol amine **(B)** concentration vs. time profile. Florfenicol was administered subcutaneously at a dose of 40 mg/kg twice 4 days apart in lactating does (*n* = 5). Florfenicol and florfenicol amine were quantified using ultra-performance liquid chromatography with tandem mass spectrometry.

Rapid residue detection testing of fresh milk samples indicated the presence of florfenicol in the milk samples of all does 12 h after each dose. The third consecutive negative RRDT strips ranged from 528 to 792 h (22 to 33 days) PSD. UPLC-MS/MS testing of fresh frozen milk samples showed that florfenicol concentrations became non-detectable earlier than the RRDT strips indicated milk samples were negative, which can be seen in Figure 3 [(A) florfenicol concentrations and (b) florfenicol amine concentrations, respectively]. Table 4 shows a comparison of milk samples time to negative between UPLC-MS/MS and RRDT strips.

**Table 4:** Sampling time point after second florfenicol treatment when florfenicol concentrations in milk samples were below the limit of detection (LOD, 3 ppb) on ultra-performance liquid chromatography with tandem mass spectrometry (UPLC-MS/MS), and after the third consecutive negative test result using the rapid residue detection test (RRDT) strips in duplicate.

**Doe N°**	**Time (h) post-second dose**		**Diff between test to Neg^a^**
	**RRDT**	**UPLC-MS/MS**	**RRDT – UPLC-MS/MS**
1	600	528	72
2	792	684	108
3	552	552	0
4	792	720	72
5	528	504	24

a Difference in hours between negative test results (below LOD of 3 ppb) using UPLC-MS/MS and the third consecutive milking that had negative test results using RRDT strips run in duplicate.

### Florfenicol and florfenicol amine in milk samples stored in the refrigerator and freezer

Rapid residue detection testing of stored refrigerated and stored frozen milk samples from the 432 h (18 days) PFD milk collection provided the same results when compared to the fresh milk collected at the same time point. However, the milk samples collected at 600 h (25 days) PFD had multiple instances where the RRDT results of the stored milk samples did not match the RRDT results completed on the fresh milk samples (Table 5). The results for milk quantification of florfenicol/amine in stored frozen milk samples for 60 days after milk collection at two time points (432 and 600 h, 18 and 25 days, respectively, PFD) are shown in Table 6.

**Table 5:** Results for the effect of freezing samples at −70°C for up to 5 days on florfenicol (ppb) and florfenicol amine (ppb) concentrations using ultra-performance liquid chromatography with tandem mass spectrometry (UPLC-MS/MS) and rapid residue detection test (RRDT) strips. Samples used on this trial were from animals in the study at specifics time points.

**Goat ID & collection time** **post-first dose^a^**	**Storage time** **post-sampling^b^**	**UPLC-MS/MS**		**RRDT (run in** **duplicate)^e^**
		**Florfenicol (ppb)^c^**	**Florfenicol amine (ppb)^d^**	
1 & 432	**Day 0**	34.84	5.13	+/+
	Day 1	32.68	6.72	+/+
	Day 3	36.32	5.28	+/+
	Day 5	23.8	7.8	+/+
1 & 600	**Day 0**	3.4	Not detected	+/+
	Day 1	34.6^*^	Not detected	+/+
	Day 3	5.48	Not detected	+
	Day 5	^∧^	^∧^	^∧^
2 & 432	**Day 0**	46.49	21.08	+/+
	Day 1	53.52	18.72	+/+
	Day 3	45.8	20.32	+/+
	Day 5	52.24	18.64	+/+
2 & 600	**Day 0**	12.41	5.28	+/+
	Day 1	11.88	5.2	+/+
	Day 3	14.72	4.44	+
	Day 5	^∧^	^∧^	^∧^
3 & 432	**Day 0**	17.57	4.49	+/+
	Day 1	17.6	5.08	+/+
	Day 3	20.52	5.36	+/+
	Day 5	17.52	8.4	+/+
3 & 600	**Day 0**	3.61	Not detected	–/–
	Day 1	4.44	Not detected	+/+^§^
	Day 3	3.44	Not detected	+/+^§^
	Day 5	Not detected	Not detected	+/–^§^
4 & 432	**Day 0**	44.08	9.32	+/+
	Day 1	41.32	10.44	+/+
	Day 3	41.28	10.48	+/+
	Day 5	36.08	9.84	+/+
4 & 600	**Day 0**	8.03	Not detected	+/+
	Day 1	8.32	2.36	+/+
	Day 3	6.72	2.8	+/+
	Day 5	6	2.2	+/+
5 & 432	**Day 0**	27.59	9.026667	+/+
	Day 1	^∧^	^∧^	^∧^
	Day 3	27.24	10	+/+
	Day 5	29.68	9.6	+/+
5 & 600	**Day 0**	Not detected	Not detected	–/–
	Day 1	Not detected	Not detected	–/–
	Day 3	Not detected	Not detected	–/–
	Day 5	Not detected	Not detected	–/–

a Goat ID number and time point post-first florfenicol dose when sample was collected from animal in the florfenicol administration trial.

b Time in days that the samples were stored. Day 0 is the reference point.

c Florfenicol concentration in milk as per UPLC-MS/MS. Limit of detection for this method is 3 ppb.

d Florfenicol amine concentration in milk as per UPLC-MS/MS. Limit of detection for this method is 3 ppb.

e RRDT results as positive (+ or +/+) for samples with either florfenicol or florfenicol amine above the detection limit, or negative (– or –/–) for samples with both florfenicol and florfenicol amine below the detection limit. RRDT detection limit is 1 ppb for florfenicol. The two values for positive (+/+) or negative (–/–) are results of testing the same sample in duplicate; two samples were not tested in duplicate.

* Sample independently analyzed in triplicate twice and confirmed. The authors believe this is an erroneous result due to an unknown cause.

∧ Sample not available for analysis.

§ Stored milk sample RRDT results that did not match the RRDT results of the fresh milk sample.

**Table 6:** Rapid residue detection test (RRDT) results and corresponding milk quantification *via* ultra-performance liquid chromatography with tandem mass spectrometry (UPLC-MS/MS) of florfenicol (florfenicol amine) in stored frozen (−70°C) milk samples for 60 days after milk collection at two time points (432 and 600 h post-first dose).

**Doe**	**Milk collection time**	**RRDT**		**UPLC-MS/MS (ppb)**	
				**Pre-storage**	**After storage**
1	^*^	^*^	^*^	^*^	^*^
2	^*^	^*^	^*^	^*^	^*^
3	600 h post-first dose	+^§^	+^§^	^∧^	^∧^
4	432 h post-first dose	+	+	^∧^	^∧^
5	432 h post-first dose	+	+	27.59 (9.03)	22.48 (7.88)
5	600 h post-first dose	–	–	ND (ND)	ND (ND)

* Not collected (due to experimental protocol changing after these time points had passed).

∧ Sample not collected/lost.

§ RRDT strips noted to be subjectively weakly positive/borderline negative.

## Discussion

Florfenicol is commonly prescribed in an extra-label manner when treating lactating dairy does despite minimal milk residue data and extrapolated goat milk withdrawal interval recommendations from cattle. Results from this evaluation indicate that a commercial RRDT validated for co-mingled cattle milk is suitable for detecting florfenicol residues in fresh milk samples from individual goats, despite the differences in milk composition between goats and cattle. Does treated with florfenicol at a dose of 40 mg/kg subcutaneously twice 4 days apart, had milk samples that remained positive on RRDT strips longer than was detectable on UPLC-MS/MS. The time to the third set of negative RRDT strips ranged from 528 to 792 h (22–33 days) PSD, whereas the time points when UPLC-MS/MS samples crossed below the LOD (3 ppb) ranged from 504–720 h (21–30 days) PSD. In addition, our results support that the RRDT manufacturer's instructions for milk samples from cattle can apply to goat milk samples, which can be stored up to 5 days in the refrigerator and 60 days in the freezer prior to testing. Lastly, our study was not able to statistically evaluate factors that could result in false positive RRDT samples, however, a trend of minimal drug degradation was observed.

Does treated with florfenicol had milk samples that remained positive on RRDT strips longer than was detectable on UPLC-MS/MS. This difference can be attributed to the difference in sensitivity between the RRDT strips and UPLC-MS/MS. The RRDT strips have a 1 ppb validated detection limit in bovine milk, while the UPLC-MS/MS LOD was 3 ppb for both florfenicol and florfenicol amine. In the only published study of subcutaneous florfenicol administration in lactating cattle, florfenicol remained above the LOQ of 5 ppb (LOD not stated) for 432–588 h (18–24.5 days) after a single 40 mg/kg dose with an associated 60 h (2.5 days) terminal elimination half-life in milk (2). The present study did administer a two-dose regimen, rather than the single dose administered in the cattle study, which may account for the longer detection time. This two-dose regimen was selected based on common clinical practice where a second dose is needed for treatment efficacy, as well as common dosing regimens submitted to FARAD. Two does in our study with milk samples that remained positive on RRDT strips longer, also had lower milk production during their lactation and lower milk fat when compared to study counterparts (Figure 2). The authors hypothesize that high milk producing animals may have an increase in the elimination of florfenicol when compared to lower producing animals. Since this study utilized healthy does, the results may not reflect overall milk production or excretion of florfenicol in unhealthy does. This is an important consideration given the known milk excretion differences of some drugs in mastitic cattle (14), which is attributed to decreased milk productions and metabolic changes.

Rapid residue detection testing of stored milk samples mostly reflected the results obtained from the testing of fresh milk samples. However, the authors noted exceptions that occurred with some of the 600 h PFD milk samples. Refrigerated 600 h PFD milk samples from multiple does either had one strip interpreted as negative and one strip interpreted as positive or visual observation indicated a subjectively weakly positive/borderline negative. Similarly, milk samples from one doe at 600 h PFD stored frozen for 2 months were noted to be subjectively weakly positive/borderline negative upon visual observation. Although the sample size of stored milk samples was small, these results seem to indicate that goat milk samples can be stored according to the RRDT manufacturer's instructions for future testing. However, caution should be exhibited when storing samples that have drug concentrations close to the sensitivity of the testing strip because our results indicate that the drug concentrations may degrade over time. This effect is shown with both the refrigerated and frozen storage samples at 600 h PFD, when the RRDT results were not in agreement for the strips run in duplicate or were subjectively fainter in color, thereby thought to be relatively close to the RRDT strip sensitivity. This effect is important to account for producers who may consider storing milk for later testing, since it would be most likely that the milk stored would be closer to the withdrawal period (and thus, lower drug concentrations closer to the testing strip sensitivity).

The use of this commercially available RRDT to detect florfenicol in individual goat milk samples provides both advantages and disadvantages as a resource to determine if milk is acceptable for sale. Since the goal of a RRDT is to determine if milk is free of drug residues prior to consumption or sale, the main advantage of this RRDT is the simple procedure required for results within 8 min. Besides the specialized incubator and RRDT strips, the remaining commercial equipment (pipets and strip-reading machine) is optional, which allows for easy setup and utilization. Another advantage is the ability to test individual goat milk samples, which could be helpful for testing milk from animals that might be outliers due to illness or low milk production. Despite these advantages, the price of both the mandatory machine and RRDT strips requires a monetary commitment. Since the manufacturer's instructions clearly explain how to interpret the RRDT the optional RRDT reading machine was not purchased for this study. However, without this RRDT reading machine, interpretation of test strip results can be subjective when milk concentrations approach analytical sensitivity. For our study, test strips were evaluated by two individuals due to the subjective nature of interpretation. Another limitation was the number of RRDT strips that resulted in being unusable (i.e., packaging was compromised, the testing well was exposed on removal from the canister, adhesive layer tearing inappropriately rendered the flap unable to close, or invalid results obtained after incubation). This combined with the high expense for the equipment makes RRDT use practical in settings where high incidences of testing are required vs. operations that would need infrequent testing. Although the RRDT can provide quick results, negative RRDT results should not be a determining factor for estimating a WDI following extra-label drug use. In addition, given the lack of currently available RRDTs validated for use with goat milk, RRDTs validated for use with cattle milk are used extra-label, so scientific validation of each RRDT according to the National Conference on Interstate Milk Shipments guidelines of 90% specificity and 90% sensitivity with 95% confidence intervals would be ideal (8, 15).

Given the small number of milk samples with milk quality and components outside of the normal range for goat milk included in this study, the potential negative effect of milk components or quality on the accuracy of RRDT results could not be assessed statistically; however, the milk components and quality parameters for the vast majority of milk samples were within manufacturer's recommended limits. The manufacturer's instructions for the RRDT used in this study indicated that certain milk components or the presence of other amphenicols may cause invalid results and potentially lead to false positives. Specifically, the manufacturer's instructions state that no interferences in detection will result from somatic cells at ≤ 10^6^ SCC/ml or bacteria at ≤ 3 × 10^5^ CFU/ml, milk fat samples (<6.5%) or other amphenicols present in concentrations <100 ppb. Some of the goat milk samples collected in this study were noted to have elevated somatic cell counts and bacterial colonies, but the impact is unclear due to a limited number of affected samples. With the exception of a single milk sample, all other milk samples had fat percentages below 6%. Future studies should be aimed to elucidate if these milk components and quality parameters affect the RRDT results by utilizing goats with and without milk components and quality parameters in the normal range.

## Conclusion

Based on comparison with UPLC-MS/MS, this study supports that the RRDT evaluated in this study can be used for detection of florfenicol residues in milk samples from individual goats treated in an extra-label manner with florfenicol. RRDT results for florfenicol residues in milk samples indicated that samples can remain positive longer than was detected using UPLC-MS/MS for nearly all goats studied. These results were likely due to sensitivity differences between the two methods. Furthermore, we also observed minimal degradation of florfenicol after storage in the refrigerator, indicating a potential use of this approach for delayed testing of goat milk for drug residues after storage. Future studies should be completed in a larger and more representative population of goats, including animals that are ill and with goats that have milk components and quality parameters outside of the normal range.

## Data availability statement

The original contributions presented in the study are included in the article/Supplementary material, further inquiries can be directed to the corresponding author/s.

## Ethics statement

The animal study was reviewed and approved by University of California Institutional Animal Care and Use Committee (IACUC; Protocol Number 21671).

## Author contributions

Conceptualization: LT, RP, and JD. Methodology: LT, RP, JD, and ER. Formal analysis: ER, JD, and RP. Data curation: RP and ER. Writing—original draft preparation: ER, LT, and RP. Writing—review and editing: JD, JR, MC, SW, and BR. Project administration: LT. All authors have read and agreed to the published version of the manuscript.

## Funding

This project was supported by the United States Department of Agriculture National Institute of Food and Agriculture for the Food Animal Residue Avoidance Databank (FARAD) Program (Award Nos.: 2020-41480-32518 and 2021-41480-35268).

## Conflict of interest

The authors declare that the research was conducted in the absence of any commercial or financial relationships that could be construed as a potential conflict of interest.

## Publisher's note

All claims expressed in this article are solely those of the authors and do not necessarily represent those of their affiliated organizations, or those of the publisher, the editors and the reviewers. Any product that may be evaluated in this article, or claim that may be made by its manufacturer, is not guaranteed or endorsed by the publisher.

## Abbreviations

ELDU: extra-label drug use
EMEA: European Medicines Agency
FDA: Food and Drug Administration
PFD: post-first dose
PSD: post-second dose
MRL: maximum residue limit
RRDT: rapid residue detection test
TOL: tolerance
UPLC-MS/MS: ultra-performance liquid chromatography with tandem mass spectrometry
WDI: withdrawal interval.
