# Supplementary material for: Comparison of florfenicol depletion in dairy goat milk using ultra-performance liquid chromatography with tandem mass spectrometry and a commercial on-farm test
Source: Front Vet Sci. 2022 Aug 29;9:991772. doi: 10.3389/fvets.2022.991772 (PMC9465015; doi:10.3389/fvets.2022.991772)
Supplement: Supplementary file 1 [file Data_Sheet_1.docx]

Comparison of Florfenicol Depletion in Dairy Goat Milk Using Ultra-Performance Liquid Chromatography with Tandem Mass Spectrometry and a Commercial On-farm Test

Emily D. Richards, PharmD^1,2^, Richard V. Pereira, BVSc, PhD^3*^, Jennifer L. Davis, DVM, PhD^1,4^, Joan D. Rowe, DVM, PhD^2^, Maaike O. Clapham^1,2^, Scott E. Wetzlich^1,2^, Benjamin A. Rupchis^5^, Lisa A. Tell, DVM^1,2*^

^1^Food Animal Residue Avoidance and Depletion Program, USA

^2^Department of Medicine and Epidemiology, School of Veterinary Medicine, University of California-Davis, Davis, CA 95616, USA

^3^Department of Population Health and Reproduction, School of Veterinary Medicine, University of California-Davis, Davis, CA 95616, USA

^4^Department of Biomedical Sciences and Pathobiology, Virginia-Maryland College of Veterinary Medicine, Blacksburg, VA 24061, USA

^5^Department of Animal Science, University of California-Davis, Davis, CA 95616, USA

***Co-Corresponding Authors:** Richard V. Pereira, and Lisa A. Tell, 1 Garrod Dr, Davis, CA, rvpereira@ucdavis.edu; latell@ucdavis.edu

Keywords: florfenicol, goat, extra-label drug use, drug residue, milk.

Supplementary Material

# Supplementary Tables

**Supplemental Table 1.** Ultra-performance liquid chromatography (UPLC) gradient method for the mobile phase used for the florfenicol analysis in goat milk samples following administration of florfenicol 40 mg/kg subcutaneously twice 4 days apart in lactating does.

| **Time**  **(min)** | **%A**  **(10mM NH4Ac+0.05% HAc)** | **%B**  **(ACN)** |
| --- | --- | --- |
| 0.00 | 87 | 13 |
| 0.25 | 87 | 13 |
| 0.75 | 2 | 98 |
| 1.25 | 2 | 98 |
| 1.26 | 87 | 13 |
| 3.25 | 87 | 13 |

%A = mobile phase A, 0 mM ammonium acetate (NH_4_Ac) + 0.05% (v/v) acetic acid (HAc) in H_2_O: %B = mobile phase B, 100% ACN.

**Supplemental Table 2.** Multiple reaction monitoring (MRM) transitions and specific mass spectrometry tuning parameters for the quantification of florfenicol and florfenicol amine in goat milk samples following administration of florfenicol 40 mg/kg subcutaneously twice 4 days apart in lactating does.

| **Analyte** | **Parent Ion**  **(amu)** | **Product Ion**  **(amu)** | **Cone Energy**  **(V)** | **Collision Energy (eV)** | **Quant/Qual Transition** |
| --- | --- | --- | --- | --- | --- |
| Florfenicol  amine | 248.1  [M+H]^+^ | 130.3 | 24 | 21 | Quantifier |
|  | 248.1  [M+H]^+^ | 230.0 | 24 | 12 | Qualifier 1 |
| Florfenicol  amine-d3  (IS) | 251.1  [M+H]^+^ | 233.1 | 24 | 12 | Quantifier |
|  | 251.1  [M+H]^+^ | 130.5 | 24 | 22 | Qualifier 1 |
| Florfenicol | 356.0  [M-H]^-^ | 185.0 | 26 | 20 | Quantifier |
|  | 356.0  [M-H]^-^ | 336.0 | 26 | 10 | Qualifier 1 |
| Florfenicol-d3  (IS) | 359.0  [M-H]^-^ | 188.0 | 28 | 20 | Quantifier |
|  | 359.0  [M-H]^-^ | 339.0 | 28 | 8 | Qualifier 1 |

**Supplemental Table 3.** Mass spectrometer tuning parameters for the detection of florfenicol and florfenicol amine in goat milk samples following administration of florfenicol 40 mg/kg subcutaneously twice 4 days apart in lactating does.

| **Parameter** | **Value** |
| --- | --- |
| Capillary (kV) | 0.50 |
| Cone (V) | 25 |
| RF (V) | 2.50 |
| Extractor (V) | 3.00 |
| Source Temperature (°C) | 150 |
| Desolvation Temperature (°C) | 600 |
| Cone Gas Flow (L/h) | 10 |
| Desolvation Gas Flow (L/h) | 1000 |

**Supplemental Table 4.** Physical examination characteristics for five dairy goat does enrolled in a florfenicol milk depletion study following administration of florfenicol 40 mg/kg subcutaneously twice 4 days apart.

| Doe | 1 | 2 | 3 | 4 | 5 |
| --- | --- | --- | --- | --- | --- |
| Breed | Alpine | Saanen | Saanen | LaMancha | Alpine x LaMancha |
| Age (yr) | 3 | 5 | 2 | 2 | 2 |
| Weight (kg) | 90 | 111.5 | 113 | 77.5 | 80 |
| BCS | 3.25 | 3.75 | 3.75 | 3 | 3.25 |
| FAMACHA® Score | 2 | 2 | 1 | 2 | 1 |
| Lactation Number | 2 | 4 | 1 | 1 | 1 |
| Days in Milk^1^ | 5 | 11 | 5 | 11 | 4 |
| Milk Production^2^ (lbs) | 7.59 ± 1.43 | 6.64 ± 1.19 | 7.59 ± 1.34 | 5.27 ± 0.84 | 5.08 ± 0.97 |

1. Days in milk for first dose of florfenicol

2. Average ± standard deviation milk production in lbs for one milking. The farm milked does twice a day.

BCS= body condition score, on a scale of 1 to 5; FAMACHA® = Faffa Malan Chart.
